# 564. Temporal External Validation of a Machine Learning Model for Sepsis Risk Prediction in Burn Patients

**DOI:** 10.1093/jbcr/irag033.304

**Published:** 2026-04-06

**Authors:** Marius Drysch, Sonja Schmidt, Felix Reinkemeier, Flemming Puscz, Marcus Lehnhardt, Christoph Wallner

**Affiliations:** University Hospital Bergmannsheil Bochum, Bochum, Nordrhein-Westfalen; BG Universitaetsklinikum Bergmannsheil, Bochum, Nordrhein-Westfalen; BG Universitaetsklinikum Bergmannsheil, Bochum, Nordrhein-Westfalen; BG Universitaetsklinikum Bergmannsheil, Bochum, Nordrhein-Westfalen; BG Universitaetsklinikum Bergmannsheil, Bochum, Nordrhein-Westfalen; BG Universitaetsklinikum Bergmannsheil, Bochum, Nordrhein-Westfalen

## Abstract

**Introduction:**

Sepsis remains a leading cause of mortality in burn patients, where the persistent post-burn hypermetabolic state may obscure early clinical signs and limit opportunities for timely intervention. To address this challenge, we previously developed a streamlined machine-learning model for sepsis prediction based on six admission-level variables (age, burned body surface area, deep partial-thickness burns, full-thickness burns, inhalation injury, and hypertension). The objective of this study was to perform a temporal external validation of this model in an independent patient cohort to confirm its robustness before clinical implementation.

**Methods:**

A retrospective analysis was performed using data from a national multicenter burn registry. The previously established admission-level model was applied to a new cohort of burn patients admitted after the original development period. Model performance was assessed for discrimination (AUC), sensitivity, specificity, and predictive values. A web-based calculator was subsequently developed to support bedside implementation.

**Results:**

In the validation cohort, the score preserved strong predictive performance with metrics closely aligned to the derivation study: AUC 0.91, sensitivity 0.81, specificity 0.85, and negative predictive value 0.98. Validation confirmed not only preserved discrimination but also reproducible patterns of feature interdependence, including non-linear effects of burn size and age on sepsis risk. These findings confirm classification accuracy across temporally distinct patient samples.

**Conclusions:**

Temporal external validation in a multicenter national registry confirmed the accuracy and stability of the previously developed sepsis prediction model in burn patients. As the first burn-specific validated predictive tool in this setting, it enables reliable early-warning risk stratification immediately upon admission.

**Applicability of Research to Practice:**

This validated score supports a development from reactive diagnosis to proactive risk management in burn care. The companion web-based calculator provides an accessible, objective tool for immediate bedside risk assessment, enabling intensified surveillance for high-risk patients (e.g., more frequent biomarker assessment, earlier advanced hemodynamic monitoring) while potentially supporting stewardship in those at low risk.

**Funding for the study:**

N/A.

